# Feasibility and Acceptability of a Self-Guided Digital Family Skills Management Intervention for Children Newly Diagnosed With Type 1 Diabetes: Pilot Randomized Controlled Trial

**DOI:** 10.2196/59246

**Published:** 2024-10-21

**Authors:** Amy Hughes Lansing, Laura B Cohen, Nicole S Glaser, Lindsey A Loomba

**Affiliations:** 1 Department of Psychological Science University of Vermont Burlington, VT United States; 2 Division of Endocrinology Department of Pediatrics University of California Davis Medical Center Sacramento, CA United States

**Keywords:** type 1 diabetes, children, family support, family dynamics, web-based intervention, feasibility, acceptability, self-guided, intervention, diabetes, RCT, randomized controlled trial, psychosocial, well-being, caregiver, communication

## Abstract

**Background:**

Family dynamics play an important role in determining the glycemic outcomes of type 1 diabetes (T1D) in children. The time interval immediately following T1D diagnosis is particularly stressful for families, and interventions to support families in adjusting their family practices to support adjustment to and management of T1D in the months following diagnosis may improve glycemic outcomes. Self-guided digital interventions offer a sustainable model for interventions to support caregivers in learning evidence-based family management skills for adjustment to and management of T1D.

**Objective:**

We hypothesized that a self-guided, web-based, family skills management program (addressing caregiver social support as well as family problem-solving, communication, and supportive behavior change strategies) initiated at the time of T1D diagnosis would improve glycemic outcomes in children with T1D. In this study, we report on the feasibility and acceptability of this program.

**Methods:**

We prospectively evaluated a sample of 37 children newly diagnosed with T1D recruited from a pediatric endocrinology clinic. Parent participants were asked to complete web-based modules addressing social support, family problem-solving, communication, and supportive behavior change strategies. Module completion was analyzed for percentage completion, patterns of completion, and differences in completion rates by coparenting status. Qualitative open-ended feedback was collected at the completion of each module.

**Results:**

A total of 31 (84%) of the 37 participants initiated the web-based program. Of those 31 participants, 25 (81%) completed some content and 15 (48%) completed all 5 modules. Completion rates were higher when coparenting partners engaged in the intervention together (*P*=.04). Of the 18 participants given a choice about the spacing of content delivery, 15 (83%) chose to have all sessions delivered at once and 3 (17%) chose to space sessions out at 2-week intervals. Qualitative feedback supported the acceptability of the program for delivery soon after T1D diagnosis. Families reported on positive benefits, including requesting future access to the program and describing helpful changes in personal or family processes for managing T1D.

**Conclusions:**

In this study, we found that a self-guided digital family support intervention initiated at the time of a child’s T1D diagnosis was largely feasible and acceptable. Overall, rates of participation and module completion were similar to or higher than other self-guided digital prevention interventions for mental and physical health outcomes. Self-guided digital programs addressing family management skills may help prevent challenges common with T1D management and can decrease cost, increase access, and add flexibility compared to traditional interventions.

**Trial Registration:**

ClinicalTrials.gov NCT03720912; https://clinicaltrials.gov/study/NCT03720912

## Introduction

Outcomes in children with type 1 diabetes (T1D) are influenced by psychosocial factors including family dynamics and parent and child psychological well-being [1]. Additionally, T1D is associated with increased child and caregiver depressive symptoms, anxiety, and burnout and decreased quality of life [2-5]. American Diabetes Association guidelines include recommendations on maintaining psychological well-being and building positive health behaviors, including health behavior counseling and psychosocial care [6]. However, it is unclear how multidisciplinary diabetes care teams can sustainably provide psychosocial education and support, especially for evidence-based family management skills for T1D management, which are typically targeted by psychologists through more intensive behavioral family therapy programs [7,8].

Moreover, the time interval immediately following T1D diagnosis is particularly stressful for families [9,10], and interventions to support families in adjusting their family practices to support adjustment to and management of T1D in the months following diagnosis may improve glycemic outcomes. For example, the child’s primary diabetes caregiver often experiences high levels of stress after diagnosis [11], and measures of diabetes distress and resilience during this early period are associated with glycemic outcomes [12]. In addition, maternal support is also known to be associated with the child’s glycemic outcomes [13]. Thus, it was theorized that early instruction for caregivers on evidence-based family management skills that support family adjustment to and management of T1D immediately following diagnosis may improve glycemic outcomes. However, access to evidence-based behavioral family therapy interventions that teach these skills are limited [14], especially in the T1D context [15,16]. Self-guided digital interventions offer a sustainable model for clinic-wide interventions to support caregivers in learning evidence-based family management skills for adjustment to and management of T1D. We conducted a prospective study to determine if self-guided, digital, evidence-based, family skills management training modules (addressing caregiver social support as well as family problem-solving, communication, and supportive behavior change strategies) initiated at the time of a child’s new diagnosis of T1D were associated with benefits in glycemic control at 1 year and 2 years after diagnosis. Here, we report on the feasibility and acceptability of this self-guided, digital, family skills management intervention. Data collection to assess the efficacy of the intervention is ongoing and will be reported on separately in the future.

## Methods

### Overview

For the full prospective study, we evaluated a sample of 74 children (ages 2-17 years) newly diagnosed with T1D. Participants were recruited through an academic hospital pediatric endocrinology clinic or during their initial hospital admission. The study was prospectively registered at ClinicalTrials.gov (NCT03720912). The sample size for this feasibility and acceptability pilot study was determined by resource availability, recruiting all eligible families from our clinic during the grant period.

### Participants

Families were eligible for participation if the child had been diagnosed with T1D within the prior 6 months. Families were excluded from the study if they were unable to complete study activities in English (n=2). Families were not specifically excluded for lack of access to the necessary technology (home computer, etc), but several families declined to enroll for this reason. This was a universal prevention program and was offered to all families who were randomized to the intervention and met the inclusion and exclusion criteria, regardless of whether they were at risk for or experiencing impaired family management skills or seeking support around family skills for adapting to T1D.

### Procedures

Eligible participants were identified by daily review of newly diagnosed patients cared for in our children’s hospital or by a review of medical records for inclusion and exclusion criteria. Eligible participants were approached in the diabetes clinic or hospital by a research coordinator who explained the study and obtained written informed consent from parents and assent from children. Parent participants completed a web-based baseline survey including measures of psychosocial outcomes and social demographics. For all families, the primary diabetes caregiver provided consent. For 2-parent households, a second parent (step or biological) that was involved in T1D care was also invited to participate and followed the same consent process.

Following the completion of their surveys, participants were randomized to the self-led digital prevention program or treatment as usual via a random draw. For families randomized to the web-based learning module group, the research staff demonstrated the REDCap (Research Electronic Data Capture; Vanderbilt University) software with the patient and family present, which included an introduction to the web-based learning module program or, if consenting remotely, the family was provided the link and verbal instruction on accessing the program. Only the primary caregiver was provided links to access the modules and only the primary caregiver provided feedback on the modules. All participants completed a follow-up survey with the same psychosocial outcomes after 12 weeks.

### Intervention

A total of 37 participants were randomized to the treatment group, which is the focus of this paper evaluating the feasibility and acceptability of the modules. Data collection for feasibility and acceptability was completed from March 2019 to September 2021. The primary caregiver was provided access to a web-based learning program with 5 modules teaching family management skills delivered via the REDCap survey platform. Each module included instructional videos and activities that took 30-45 minutes to complete. Each module focused on a specific family management skill as applied in the context of helping the parents and the child adjust to managing T1D, including increasing social support for parents, collaborative problem-solving skills for navigating T1D-related challenges, communication skills for T1D management, and supportive behavior change strategies for helping children adjust to T1D management tasks (Textbox 1). The fifth module reviewed the core content and provided advice on application of skills in T1D contexts.

**Overview of intervention content. Parent participants were provided access to 5 self-guided, digital, evidence-based modules teaching family management skills relevant to navigating newly diagnosed type 1 diabetes (T1D). Each module focused on a specific skill and included instructional videos and activities.**

**Introduction module**

- Overview of the program

**Module 1: social support and goal setting**

- Building a healthy social support network
- Knowing when and how to ask for help
- Setting SMART (specific, measurable, achievable, relevant, and time-bound) goals with social support

**Module 2: problem-solving**

- Introduction to problem-solving
- Defining the problem
- Brainstorming solutions
- Evaluating solutions
- Creating a plan
- Evaluating the plan

**module 3: communication strategies**

- Active listening
- Speaking effectively with “I” statements
- Negative communication traps and positive strategies
- Assertive communication

**Module 4: behavior change**

- Strategies for supporting healthy habits
- Effective praise in behavior change
- Behavioral contracts for positive behavior change

**Module 5: applying skills to T1D**

- Social support and shared goals
- Problem-solving and communication skills
- Supportive behavior change strategies
- Family management skills
- Reaching out for help

Families were encouraged to do a module at a standard interval of 1 module every 2 weeks but could pace themselves to complete them at any time in the 12-week window. Modules were required to be completed by 1 parent, but parents were encouraged to work with a care partner of choice. Primary caregivers received automated SMS text message and email reminders to complete the modules. Technical support from research staff was available to participants via telephone and email, but no participants sought technical support. Each module ended with an inquiry regarding if the module was completed alone or with a partner and the relationship to this person (eg, spouse, friend, or sibling), with an option to provide open-ended feedback on the module.

### Measures

#### Program Completion

The completion of each module was tracked and time stamped as the parents completed participation. Start and completion dates and times were recorded. The percentage of parents completing each module and the total number of modules completed were calculated based on completion time stamps. In addition, parents could request to do the next module at a future date or do the next module right away, and this decision was also automatically recorded. The data were coded to identify the frequency that parents completed the modules: all in 1 sitting versus spread out across the 12 weeks.

#### Parent Feedback on Modules

At the end of each module, parents were asked an open-ended question about their experience with the module. The question read, “We would love your feedback on this module to help us develop better interventions for the future. Please provide any questions, comments, suggestions, or complaints you have about the videos, the question prompts, scheduling, or completing future modules.” Full answers were recorded in the survey system (REDCap) and retained for data analysis. Participant feedback was reviewed, and a coding dictionary was created by 2 research assistants who were not involved in the consent process for participants. Each research assistant then coded the responses, and any coding discrepancies were resolved by the first author. Feedback themes were compiled across modules.

#### Family Structure and Participation

Parents reported whether or not they were married, and then further information was provided narratively on the structure of the family (married to biological parent of the child, step-parent household, or other care providers in the household). These data were coded to classify 2 types of family structure that described access to an in-household support partner: married or coparenting couple versus single parent. Parents also reported if they had a partner participate in the intervention modules.

### Ethical Considerations

The study was approved by the University of California, Davis Institutional Review Board (which serves as the local ethics committee for human research; reference 1303325-15). Informed consent was obtained from all participants. To facilitate follow-up within the confines of the study, the database was not deidentified. All data were stored securely in REDCap and only deidentified data are reported. A US $50 gift card was provided to all families as an enrollment incentive. Compensation was not tied to completing the intervention modules for those randomized to the intervention to test the feasibility and acceptability of this self-led digital dissemination model as it would be accessed in real-world contexts.

## Results

### Participants

**Figure 1:**
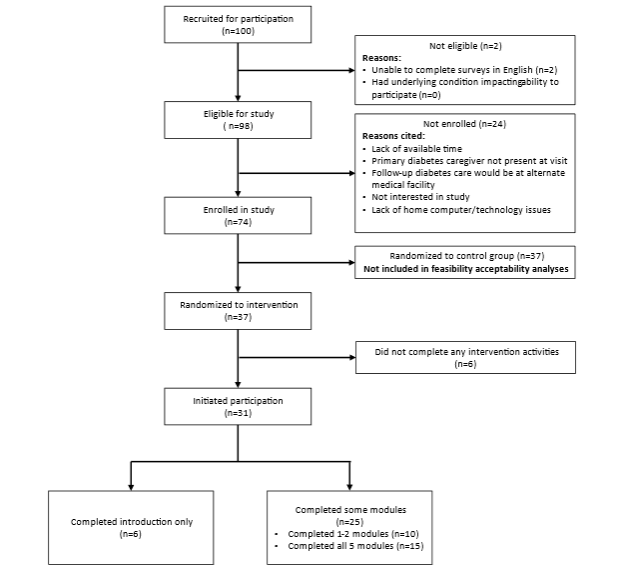
CONSORT (Consolidated Standards of Reporting Trials) flowchart.

Participants receiving the self-guided intervention included 37 parents of a child newly diagnosed with T1D (Table 1). A CONSORT (Consolidated Standards of Reporting Trials) flowchart detailing the progress of the study participants through the trial is provided in Figure 1.

**Table 1:** Demographics of the study population (n=37).

Demographics			Value
**Child’s demographics**			
	Age (years), mean (SD; range)	9.33 (3.85; 2-17)	
	Age at diagnosis (years), mean (SD; range)	9.28 (3.86; 2-17)	
**Parent’s race and ethnicity, n (%)^a^**			
	White	28 (76)	
	Asian	2 (5)	
	Black	0 (0)	
	Native Hawaiian or Pacific Islander	1 (3)	
	Hispanic or Latino	4 (11)	
	More than 1 race	1 (3)	
	Unknown or not reported	1 (3)	
	Total	37 (100)	
**Parent’s relationship status, n (%)^a^**			
	Married	25 (67)	
	Divorced	2 (5)	
	Separated	0 (0)	
	Not married	6 (16)	
	Remarried	1 (3)	
	Romantic partner	3 (8)	

^a^Parent’s relationship status and race and ethnicity were self-identified by parent participants via a web-based baseline survey following study enrollment.

### Feasibility

Our first aim was to assess the feasibility of this digital and universal T1D family management skills program in a real-world context (ie, without incentives to participate). First, we examined descriptive statistics for the percentage of modules completed. Second, we examined what percentage of participants completed modules all in 1 sitting or across multiple weeks. Third, we evaluated differences in module completion rates by coparenting status, that is, whether the primary parent was in a coparenting couple or married versus a single parent, and for those in a coparenting couple or who are married, if module completion rates differed by whether the parenting partner also participated in the program.

Out of a total 37 participants randomized to receive the intervention, 31 (84%) initiated participation in the program by completing the introduction overview session. Of those 31 participants, 6 (19%) completed the introduction overview only, 10 (32%) completed 1-2 additional modules, and 15 (48%) completed all 5 additional modules. All participants who went beyond 2 modules completed the full program.

In addition, of the 25 participants that completed at least some intervention content beyond the introduction overview session, 18 (72%) were given a choice about the spacing of the sessions and 7 (28%) had sessions spaced out every 2 weeks without being given the choice to complete all sessions at once. This variation was the result of a change in study protocol after the first 7 participants, many of whom requested to have the option to complete the modules all at once. Of the 18 participants given a choice about the spacing, 15 (83%) chose to have all sessions delivered at once and 3 (17%) chose to space sessions out at 2-week intervals. There was no significant difference in the number of sessions completed according to the timing of session delivery overall (*F*_2,22_=1.29; *P*=.30).

Finally, of the 31 participants that initiated participation in the intervention, 25 (81%) reported being married to a parenting partner or living with a parenting partner who helped with managing their child’s T1D, and 6 (19%) reported not living with a parenting partner who helped with managing their child’s T1D. There was no difference in the number of modules completed according to access to a parenting partner (*t*_9.20_=–1.78, equal variances not assumed; *P*=.11). Of the 25 parents who had access to a parenting partner, 21 (84%) reported on whether their partner engaged with them in the modules; of these 21 participants, 12 (57%) reported that their partner participated in the learning modules and 9 (43%) reported that their partner did not participate in the learning modules. There was a significant difference in module completion between these 2 groups (t_19_=–1.89; *P*=.04), with parents who completed the modules with a partner completing more modules (mean 3.92, SD 2.02) than those whose parenting partner did not do the modules (mean 2.11, SD 2.26). Further, parents whose partners did not engage in the modules reported similar completion rates as parents without a parenting partner (mean 1.67, SD 1.75).

### Acceptability

A qualitative thematic analysis assessing participant feedback with the program was completed to assist in assessing acceptability and revising the program for future studies. Open-ended responses could be provided after each module but were not required as part of study participation. A total of 13 participants chose to provide feedback during participation. Five themes were identified: general helpfulness (n=6), specific positives (n=6), specific challenges (n=6), neutral feedback (n=3), and poor fit for family (n=2). Detailed feedback responses from each theme are provided in Multimedia Appendix 1.

First, although the open-ended question did not indicate a specific request for positive feedback, the majority of feedback involved descriptions of (1) the general helpfulness of the program, that is, learning to work as a family to manage T1D and feeling seen in challenges that the families were navigating after the diagnosis of T1D, as well as (2) specific positive features of the modules, that is, the way modules generated conversations with a partner and were easy to follow. Second, the open-ended question did directly ask for suggestions for improving the modules, and families suggested (1) increasing accessibility, that is, adding transcripts and better sound quality, as well as phone presentation, and tools to help focus; (2) improving the system so prior work can be more readily accessed; (3) offering more content specifically for people with split households; and (4) allowing people to access repeatedly in the future. Third, 3 participants offered neutral feedback that they had no questions, suggestions, or comments. Last, only 2 participants described the program as a poor fit for their family, such that their family was not facing challenges with T1D management and that they could not relate to the content provided.

## Discussion

In this study, we found that a self-guided, digital, family support intervention initiated at the time of a child’s T1D diagnosis was largely feasible and acceptable. This intervention was designed to assist the primary diabetes caregiver (typically the child’s mother) with family management skills that are associated with more positive outcomes in children with T1D, including accessing social support, collaborative problem-solving, family communication, and supportive child behavior change strategies. Although the modules offered instruction on these skills tailored to the T1D context, they did not specifically target instruction on diabetes self-management tasks. This program was delivered in a way that was consistent with a clinic-wide, opt-out format, that is, all families with a new diagnosis of T1D were invited to participate in the study regardless of psychosocial treatment–seeking status or selected status for higher risk (ie, only enrolling families that requested more support or that had multiple psychosocial or structural risk factors for challenges with managing T1D). Further, consistent with expectations of limited funding resources if disseminated, there was no financial incentive offered to participants randomized to the intervention to incentivize module completion.

First, rates of participation and module completion in this study were similar to or higher than other self-guided, digital prevention interventions for mental and physical health outcomes, suggesting that this program is feasible for clinic-wide, opt-out delivery. Further, 15 (41%) of our 37 participants completed all the program content, whereas meta-analyses of self-guided digital interventions for depression suggest an adherence rate of 26% in a treatment-seeking sample [17] and between 1% and 26% in broader mental health samples [18]. In a meta-analysis of self-guided digital interventions for treatment-seeking patients with chronic health conditions, 41% to 45% completed the programs [19]. Many of these programs also offered incentives for completing the study intervention, which were not offered in our model. Similarly, after randomization, 84% (31/37) of participants initiated participation in our modules, and this was consistent with other larger self-guided digital intervention trials with larger samples (eg, 747/947, 79% of participants initiated participation in a self-guided, web-based, mental health intervention) [20]. Finally, of those who initiated treatment in the intervention, 48% (15/31) completed all 5 modules and 81% (25/31) completed at least some content.

Moreover, 19% (6/31) completed no content after completion of the introduction, and our qualitative data indicate that these participants did not identify a need for additional support on the skills taught. This is again consistent with prior data on non–treatment-seeking individuals, and overall, our engagement rates were higher than rates of participation in other family management skills models and similar to rates in effectiveness trials for family and child therapy. For example, in a universal prevention program, only 17% of eligible families attended at least 1 family intervention session [21], while other studies have suggested lower rates of participation in family models [22]; dropout rates in effectiveness studies for family therapy average around 50% [23].

Second, qualitative feedback also supported that this self-guided web-based program was largely acceptable to families for delivery soon after a new T1D diagnosis. Families consistently reported on the positive benefits of the intervention, including requesting further access to the program in the future and describing helpful changes in their personal or family processes for managing T1D. In addition, we found that this program was more acceptable when coparenting partners engaged in the intervention together, that is, higher completion rates for people who participated in the modules with a coparenting partner compared to those whose partner did not participate and those who did not have a partner. One family provided feedback that more content focused on split household families was needed, so future iterations of this intervention should include additional content for this family context to increase acceptability. It might also be useful to tailor a version for specific family contexts by integrated evidence-based practices from research on helping primary caregivers with limited, complicated, or high-conflict support networks/coparents. Finally, one family noted that exhaustion in the postdiagnosis phase limited their ability to focus on the intervention content, while another wanted written transcripts of the content. Thus, it may be important to revise the intervention content for greater accessibility across cognitive contexts following universal learning methods.

In summary, self-guided digital tools can decrease cost, increase access, and add flexibility for chronic disease management [19,24,25]. The modules used in this study were completed at personally convenient times, were self-paced, and posed no risk of further lost work or wages for parents already dealing with the effects of a child’s recent hospitalization. The low cost of production and support to access the modules make them scalable for resource-limited practices. In addition, although practices may not have the time or funds to offer intensive instruction in family management skills at the time of diagnosis, a self-guided digital program may help prevent the onset of challenges with T1D management as the honeymoon period ends and as many newly diagnosed families move further into adolescence. Further follow-up data collection and analysis are ongoing to examine the effectiveness of this intervention on longer-term family and glycemic outcomes.

## Appendix

Multimedia Appendix 1 Themes and responses from open ended questions soliciting feedback.

Multimedia Appendix 2 CONSORT-eHEALTH checklist (V 1.6.1).

## Abbreviations

CONSORT: Consolidated Standards of Reporting Trials
REDCap: Research Electronic Data Capture
T1D: type 1 diabetes

## Data Availability

All data generated or analyzed during the feasibility and acceptability portion of this study are included in this published paper and its supplementary files. The datasets generated during the greater study on glycemic outcomes are not publicly available as data collection is ongoing but are available from the corresponding author on reasonable request.
