# Supplementary material for: Feasibility and Acceptability of a Self-Guided Digital Family Skills Management Intervention for Children Newly Diagnosed With Type 1 Diabetes: Pilot Randomized Controlled Trial
Source: JMIR Form Res. 2024 Oct 21;8:e59246. doi: 10.2196/59246 (PMC11535796; doi:10.2196/59246)
Supplement: Multimedia Appendix 1 [file formative_v8i1e59246_app1.docx]

Multimedia Appendix 1. Themes and responses from open ended questions soliciting feedback (n=13).

| **Themes** | **Comments** |
| --- | --- |
| **General Helpfulness**  Participants described that they generally found program helpful  N=6 | “It was helpful 😊”  “This whole series was awesome… I have already shared the impacts of participating in these modules… my whole approach to diabetes management and my support network were changed for the better because of participating in these modules.”  “This is thoughtful and helpful content. Thank you for offering [resources]. I feel seen and it helps to know my feelings are valid.”  “This was a very helpful module once we were able to sit and focus.”  “I really do think these modules have helped. A lot of it seems super obvious, but are all things that we definitely needed to hear again to be able to implement.”  “Thank you so much, this was very helpful for my son and I.” |
| **Specific Positives**  Participant described specific things they liked about the modules or program  N=6 | “Definitely enjoy that there’s clear instructions on how to walk through something step by step.”  “I liked [the] conversations this one started [with my partner].”  “The video was to the point and explained what we could expect.”  “Thank you for… creating a space where I can generate reflection and reconsider communication patterns with my spouse.”  “Each module did a great job of breaking things down into understandable and achievable goals.” |
| **Specific Challenges**  Participant described specific things they recommended to have change about the program or found challenging with the program  N= 6 | “Offering a transcript of videos, I have a hard time focusing on listening at times and would like to read”  “We started out rocky on this one…we have been a little sleep deprived lately, so a little short focused.”  “A suggestion is to fix the form when filling out on a phone.”  “It is difficult to remember all prior answers from previous modules. [Can you make] those available to us in this site.”  “Mentioning that we should just work on one problem at a time and that others should “wait” seemed out of touch. In reality, we are now dealing with a chronic disease and some of these problems need to be solved quickly. Perhaps you could suggest that each partner take a few.”  “For split households and like mine unfortunately communication is not great, I [am] not sure of a solution; however, [I need] something in that area.”  “It would also be helpful to repeat them because goals and caring for a type 1 diabetic changes every year.”  “The sound quality was poor.” |
| **Neutral Feedback**  Participants noted they had no feedback.  N=3 | “So far, no complaints, suggestions, or comments.”  “Don’t have any questions.” |
| **Poor Fit for Family**  Participants described that they did not find program helpful or the right fit for their family.  N= 2 | “The modules seem to be focusing more on problems. Makes me feel we’re supposed to be struggling more than we are.”  “I am trying to complete this study, but this really doesn’t apply to our family.” |

*13 participants provided qualitative feedback on their experience with the modules. Five themes were identified: general helpfulness (n=6), specific positives (n=6), specific challenges (n=6), neutral feedback (n=3), and poor fit for family (n=2).*
